# Cell Type‐Specific mTORC1 Signaling and Translational Control in Synaptic Plasticity and Memory

**DOI:** 10.1111/jnc.70281

**Published:** 2025-11-14

**Authors:** Ziying Huang, Niaz Mahmood, Shane Wiebe, Arkady Khoutorsky, Jean‐Claude Lacaille, Nahum Sonenberg

**Affiliations:** ^1^ Department of Biochemistry McGill University Montréal Quebec Canada; ^2^ Goodman Cancer Institute Montréal Quebec Canada; ^3^ Department of Anesthesia McGill University Montréal Quebec Canada; ^4^ Department of Neurosciences, Center for Interdisciplinary Research on Brain and Learning (CIRCA) and Research Group on Neural Signaling and Circuitry (GRSNC) Université de Montréal Montréal Quebec Canada; ^5^ Integrated Program in Neuroscience, Montréal Neurological Institute McGill University Montréal Quebec Canada

**Keywords:** excitatory neurons, glia, interneurons, memory, mTORC1, synaptic plasticity, translation

## Abstract

Synaptic plasticity and memory formation require *de novo* protein synthesis. The mechanistic/mammalian target of rapamycin complex 1 (mTORC1) promotes mRNA translation initiation in the central nervous system. Recent research has uncovered that excitatory neurons, inhibitory neurons, and glia play distinct roles in modulating synaptic strength and encoding long‐term memory via mTORC1 signaling. In this review, we discuss the mechanisms by which mTORC1 regulates translation initiation in the brain and its cell type‐specific roles in shaping distinct forms of synaptic plasticity and memory. We also consider how dysregulated translational control contributes to neurological disorders and explore emerging technologies for therapeutic modulation of the mTORC1 pathway.

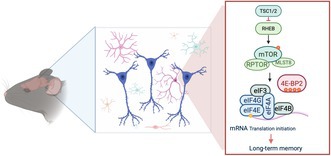

## Graphical abstract

The formation of long‐term memories depends on *de novo* protein synthesis, a process tightly regulated by the mechanistic/mammalian target of rapamycin complex 1 (mTORC1) signaling pathway. mTORC1 plays a central role in promoting mRNA translation in the central nervous system. Emerging evidence suggests that distinct brain cells, including excitatory neurons, inhibitory neurons, and glial cells, utilize mTORC1 signaling in a cell type‐specific manner to modulate synaptic plasticity and memory. This review summarizes the molecular mechanisms by which mTORC1 regulates translation initiation in various brain cell types and its contributions to different forms of synaptic plasticity and memory. Additionally, it presents novel approaches for investigating cell type‐specific translation and discusses the implications of dysregulated mTORC1 signaling in neurological disorders.

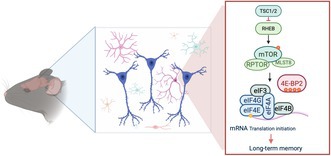

**Abbreviations**
4E‐BP: eukaryotic initiation factor 4E‐binding protein
AAV: adeno‐associated virus
AD: Alzheimer's disease
AKT/PKB: AKT/protein kinase B
AMPAR: α‐amino‐3‐hydroxy‐5‐methyl‐4‐isoxazolepropionic acid receptor
AMPK: AMP‐activated protein kinase
ASD: autism spectrum disorder
CAMKII: calcium/calmodulin‐dependent protein kinase II
CHAT: choline acetyltransferase
cKO: conditional knockout
CNS: central nervous system
DREADDs: designer receptors exclusively activated by designer drugs
eIF4E: eukaryotic initiation factor 4E
eIF4G: eukaryotic initiation factor 4G
E‐LTP: early‐LTP
EMX1: empty spiracles homeobox 1
ERK: extracellular signal‐regulated kinase
FKBP12: FK506‐binding protein 12
FMRP: fragile X messenger ribonucleoprotein 1
FXS: Fragile X syndrome
GABA: gamma‐aminobutyric acid
GAD2: glutamate decarboxylase 2
hKO: heterozygous knockout
KO: knockout
LARP1: La‐related protein 1
L‐LTP: late LTP
LTD: long‐term depression
LTM: long‐term memory
LTP: long‐term potentiation
MAPK: mitogen‐activated protein kinase
MEK: mitogen‐activated protein kinase kinase
mEPSCs: miniature excitatory postsynaptic currents
mGluR: metabotropic glutamate receptor
MNK: mitogen‐activated protein kinase‐interacting kinase
mTORC1: mechanistic/mammalian target of rapamycin complex 1
mTORC2: mechanistic/mammalian target of rapamycin complex 2
NMDAR: N‐methyl‐D‐aspartate receptor
PABP: poly(A)‐binding protein
PCs: Purkinje cells
PD: Parkinson's disease
PI3K: phosphoinositide 3‐kinase
PIC: pre‐initiation complex
PTEN: phosphatase and tensin homolog
PV: parvalbumin
RHEB: RAS homolog enriched in brain
RPTOR: regulatory associated protein of mTOR
S6K: ribosomal protein S6 kinase
scRNA‐Seq: single‐cell RNA sequencing
SST: somatostatin
STM: short‐term memory
TRAP: translating ribosome affinity purification
TSC: tuberous sclerosis complex
VGLUT1: vesicular glutamate transporter 1
VIP: vasoactive intestinal peptide

## 1 Introduction

Memory formation requires coordinated neuronal processes across multiple brain regions and circuits. At the cellular level, memory is encoded through activity‐dependent changes in synaptic strength, a phenomenon known as synaptic plasticity. Long‐term potentiation (LTP) and long‐term depression (LTD), the canonical forms of synaptic plasticity, represent candidate cellular mechanisms underlying learning and memory (Bliss and Gardner‐Medwin 1973; Choi and Kaang 2022). Enduring changes in synaptic strength require *de novo* protein synthesis, specifically through mRNA translation, which involves the stages of initiation, elongation, and termination. Among these, translation initiation is a highly regulated step, controlled by multiple intracellular signaling pathways (Kandel 2001; Klann and Sweatt 2008). The mRNA cap‐binding protein eukaryotic initiation factor 4E (eIF4E) is a critical factor in translation initiation, and its activity is modulated by mechanistic/mammalian target of rapamycin (mTOR) and mitogen‐activated protein kinase (MAPK)‐interacting kinases (MNKs). eIF2α phosphorylation can also selectively regulate translation initiation of mRNAs involved in synaptic function and plasticity (Ho‐Tieng et al. 2025; Trinh and Klann 2013). For instance, mTOR complex 1 (mTORC1)‐mediated control of translation initiation within excitatory neurons, inhibitory interneurons, and glia controls memory‐related plasticity and the diversity of associated behavioral phenotypes across distinct brain regions (Artinian et al. 2019; Khlaifia et al. 2022). These differences may underlie age‐dependent changes in cellular signaling, variations in activity‐dependent responses, and cell type‐specific forms of plasticity (Chaves et al. 2024; Gray and Spiegel 2019; Huang et al. 2024; Majumder et al. 2023). Recent discoveries have been driven by advances in genetic engineering, such as conditional knockout (cKO) mouse models and gene expression profiling, such as RNA‐Seq and Ribo‐Seq.

In this review, we provide an overview of the mTORC1 signaling pathway and how it regulates cap‐dependent translation initiation. We focus on how this pathway modulates synaptic plasticity and memory formation both circuit‐wide and through distinct mechanisms in specific cell types. We discuss emerging technologies that enable the precise dissection of mRNA translation control in vivo. These insights offer a novel perspective on how dysregulated protein synthesis contributes to cognitive disorders and inform the development of potential therapeutic interventions.

## 2 Overview of Translation Initiation and Its Link to Memory Formation

**FIGURE 1:**
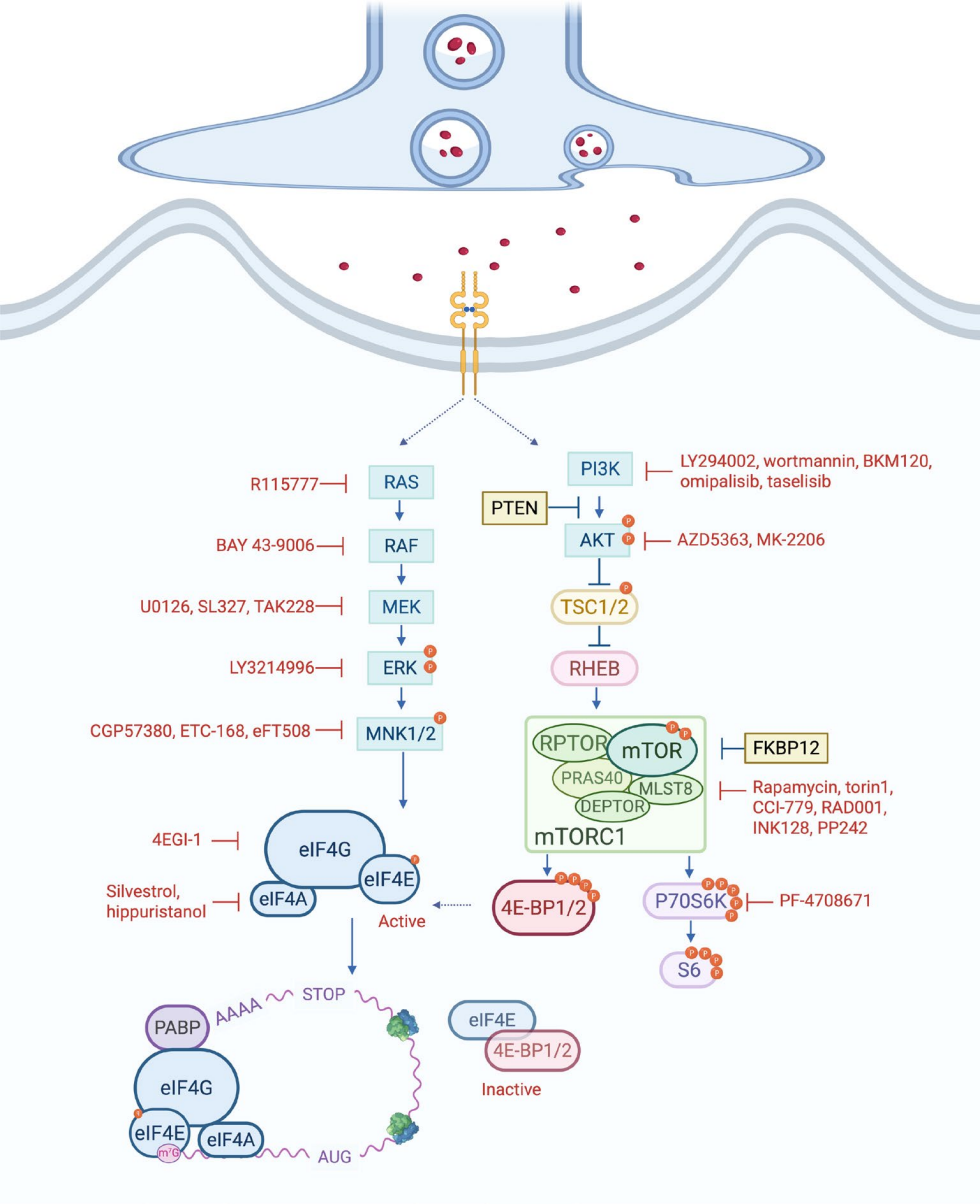
Translational control in the brain via mTORC1 signaling and eIF4E‐dependent translation. Schematic of the core pathways regulating eIF4E‐dependent translation initiation in the brain, emphasizing upstream regulation by the mTORC1 and ERK pathways. mTORC1 controls translation by phosphorylating 4E‐BP2 and releasing its repression of eIF4E, and by modulating S6 activity. The ERK pathway promotes the translation of specific subsets of mRNA via MNK1/2‐mediated phosphorylation of eIF4E. Initiation of translation involves the formation of the eIF4F complex, consisting of eIF4E (a cap‐binding protein), eIF4G (a scaffolding protein), and eIF4A (an RNA helicase), at the mRNA's 5′ cap. Pharmacological inhibitors of the PI3K/AKT/mTORC1 and RAS/ERK pathways, which are commonly employed in translational neuroscience research to modulate protein synthesis and investigate disease mechanisms, are also shown. 4E‐BP2, eIF4E binding protein 2; AKT, protein kinase B; DEPTOR, DEP domain‐containing mTOR interacting protein; eIF4F, eukaryotic initiation factor 4F; ERK, extracellular signal‐regulated kinase; FKBP12, FK506‐binding protein 12; MEK, MAPK/ERK kinase; MLST8, mammalian lethal with SEC13 protein 8; MNK1/2, MAPK‐interacting kinase 1/2; PABP, poly(A)‐binding protein; PI3K, phosphoinositide 3‐kinase; PRAS40, proline‐rich AKT substrate of 40 kDa; PTEN, phosphatase and tensin homolog; RAF, Raf proto‐oncogene, serine/threonine kinase; RAS, RAS proto‐oncogene, GTPase; RHEB, RAS homolog enriched in brain; RPTOR, regulatory associated protein of mTOR; S6, ribosomal protein S6; S6K, ribosomal protein S6 kinase; TSC1/2, tuberous sclerosis complex 1/2.

Precise translational control is critical for synaptic plasticity and long‐term memory (LTM) formation, enabling neurons to rapidly synthesize proteins in response to synaptic signals. Translation initiation is widely recognized as the rate‐limiting and most tightly regulated step in mRNA translation (Hershey et al. 2018). It commences with the assembly of the 43S pre‐initiation complex (PIC), comprising the 40S ribosomal subunit, the ternary complex (eIF2–GTP–Met‐tRNAᵢ), and initiation factors including eIF1, eIF1A, eIF3, and eIF5. The PIC's recruitment to the 5′ end of an mRNA is mediated by the eIF4F complex, which recognizes the five‐prime cap (5′ cap)—a methylated guanine nucleotide (m7G) that protects the mRNA and promotes translation. The eIF4F complex consists of the cap‐binding protein eIF4E, the RNA helicase eIF4A, and the scaffold protein eIF4G, and facilitates mRNA scanning and translation initiation codon recognition to form the 80S initiation complex (Amiri et al. 2025). eIF4E's activity is tightly controlled by mTORC1 and extracellular signal‐regulated kinase (ERK) pathways (Figure 1; Sonenberg and Hinnebusch 2009). eIF4E‐binding proteins (4E‐BPs) inhibit eIF4E by binding to it and blocking its interaction with eIF4G. mTORC1‐mediated phosphorylation of 4E‐BPs reduces their binding affinity for eIF4E, thereby promoting the formation of the eIF4F complex and stimulating translation. MNK1/2, activated downstream of ERK, phosphorylate eIF4E at Ser209 to modulate its activity and translation initiation (Sonenberg and Hinnebusch 2009).

The requirement for protein synthesis in LTM was demonstrated six decades ago using the global protein synthesis inhibitor puromycin in goldfish and mice (Agranoff and Klinger 1964; Barondes and Cohen 1966; Flexner et al. 1963, 1965). It was administered either systemically or directly into the brain, leading to memory deficits across various behavioral paradigms. Subsequent work revealed that local synaptic translation supports activity‐dependent plasticity, prompting interest in the pathways that regulate neuronal mRNA translation (Kang and Schuman 1996; Tang et al. 2002). Many studies have demonstrated that eIF4E‐dependent translation pathways are crucial for LTM formation (Gindina et al. 2021; Hoeffer et al. 2011; Santini et al. 2014). In particular, mTORC1 has emerged as a central regulator of protein synthesis during memory consolidation and reconsolidation, including in recognition, associative, and spatial memory (Bramham et al. 2016; Costa‐Mattioli et al. 2009; Gkogkas et al. 2010; Graber et al. 2013; Hoeffer and Klann 2010; Santini et al. 2014; Wiebe et al. 2020). Although the three major cell types in the brain—excitatory neurons, inhibitory neurons, and glia—are known to contribute differentially to memory processes, the field has only recently begun to systematically reveal the cell type‐specific mechanisms regulating translation.

## 3 Translational Regulation by mTORC1 Signaling in Memory

### 3.1 Upstream Regulators and Downstream Effectors of mTORC1 Signaling

#### 3.1.1 mTOR Complexes: Components and Functions

Originally identified as the target of the immunosuppressant rapamycin in yeast, mTOR is a serine/threonine kinase conserved across eukaryotes (Brown et al. 1994; Heitman et al. 1991; Sabatini et al. 1994; Stan et al. 1994). In mammals, mTOR is part of two functionally distinct multiprotein complexes: mTORC1 and mTORC2 (Loewith et al. 2002; Sabers et al. 1995). The complexes are defined by specific adaptor proteins: regulatory‐associated protein of mTOR complex 1 (RPTOR) in mTORC1 and RPTOR‐independent companion of mTOR complex 2 (RICTOR) in mTORC2 (Loewith et al. 2002; Sarbassov et al. 2004; Jacinto et al. 2004). In addition to mTOR, these distinct complexes share common components such as mTOR‐associated protein MLST8 (G protein β‐subunit‐like/GBL), and DEP domain containing mTOR interacting protein (DEPTOR) (Fonseca et al. 2007; Hara et al. 2002; Kim et al. 2002, 2003).

mTORC1 is rapamycin‐sensitive and primarily localizes to the surface of lysosomes, where it integrates signals related to proliferation, nutrient levels, and energy status (Brown et al. 1994; Fonseca et al. 2007; Stan et al. 1994; Thoreen et al. 2009). mTORC1 controls translation primarily through phosphorylation of two key substrate families: 4E‐BPs and the ribosomal S6 kinases (S6Ks) (Figure 1; Hay and Sonenberg 2004; Mutvei et al. 2020; Priya et al. 2023). Conversely, mTORC2 is largely rapamycin‐insensitive and regulates cytoskeletal organization and metabolism through phosphorylation of downstream kinases, including protein kinase B (PKB/AKT), protein kinase C (PKC), and serum/glucocorticoid‐regulated kinase (SGK) (Cameron et al. 2011; Guertin et al. 2006; Jacinto et al. 2004; Lu et al. 2011; Thoreen et al. 2009). However, prolonged exposure to rapamycin disrupts mTORC2 in some tissues and cell types (Lamming et al. 2012; Sarbassov et al. 2004, 2006).

mTORC1 and mTORC2 are functionally interconnected through multiple regulatory mechanisms that form a feedback circuit. mTORC2 promotes mTORC1 activity by phosphorylating AKT (at Ser473), which in turn enhances phosphorylation of AKT (at Thr308), thus promoting mTORC1 signaling (Baffi et al. 2021; Sarbassov et al. 2006, 2005; Yang et al. 2015). While mTORC1 and mTORC2 signaling have been extensively characterized in non‐neuronal systems, their organization and functional dynamics in different neuronal and glial subtypes remain an active area of investigation.

#### 3.1.2 Upstream Regulators of mTORC1

mTORC1 regulates protein synthesis by integrating diverse extracellular and intracellular signals, including growth factors, amino acid availability, energy levels, and oxygen status (Hay and Sonenberg 2004), to modulate brain functions.

The phosphatidylinositol‐3‐kinase (PI3K)–AKT axis is activated upon growth factor stimulation (Figure 1). PI3K converts phosphatidylinositol‐4,5‐bisphosphate into phosphatidylinositol‐3,4,5‐trisphosphate, which facilitates the recruitment of AKT to the plasma membrane (Alessi et al. 1997; van Slegtenhorst et al. 1998; Vander Haar et al. 2007). AKT, activated by PI3K‐dependent kinase and mTORC2, phosphorylates and inhibits tuberous sclerosis complex (TSC)1/2, GTPase‐activating proteins for RAS homolog enriched in brain (RHEB), promoting RHEB‐GTP accumulation and mTORC1 activation (Long et al. 2005; Sancak et al. 2008; Stocker et al. 2003; Tee et al. 2003).

The MAPK/ERK cascade is activated by RAS in response to diverse signals, including growth factors, G protein‐coupled receptors, and chemokines (Ayush et al. 2016; Della Rocca et al. 1999; Luttrell et al. 2001; Venkatakrishnan et al. 2000; Xing et al. 1996). It enhances mTORC1 activity via p90 ribosomal S6 kinase‐mediated phosphorylation of TSC2 (at Ser939 and Thr1462) and direct phosphorylation of RPTOR (at Ser696 and Ser863), boosting 4E‐BP1‐dependent translation (Carriere et al. 2011; Ma et al. 2005; Mendoza et al. 2011; Roux and Blenis 2004; Roux et al. 2007).

Additional upstream regulators of mTORC1 that act through TSC1/2 include: AMP‐activated protein kinase (AMPK), regulated in development and DNA damage response 1 (REDD1), glycogen synthase kinase 3 (GSK3), and the Wnt signaling pathway. Under low ATP conditions, AMPK phosphorylates TSC2 (at Thr1227 and Ser1345) and RPTOR (at Ser722 and Ser792), promoting 14–3‐3 binding and inhibiting mTORC1 (Gwinn et al. 2008; Inoki et al. 2003; Kimura et al. 2003). In hypoxia, REDD1 activates TSC1/2 and inhibits AKT via protein phosphatase 2A, further repressing mTORC1 (Dennis et al. 2014; DeYoung et al. 2008; Kim et al. 2023; Sofer et al. 2005). Wnt signaling inhibits GSK3 in neurons, leading to decreased TSC2 activity and subsequent mTORC1 activation (Inoki et al. 2006; Ma et al. 2011; Urbanska et al. 2018).

#### 3.1.3 Downstream Targets of mTORC1

mTORC1 regulates translation primarily by phosphorylating 4E‐BPs to control cap‐dependent initiation and S6K1/2 to control both initiation and elongation (Figure 1; Hay and Sonenberg 2004). mTORC1 substrates, including the 4E‐BPs and S6Ks, bind RPTOR via the TOR signaling (TOS) motif, enabling their phosphorylation by mTORC1 in a RHEB‐dependent manner (Long et al. 2005; Schalm and Blenis 2002; Tee et al. 2003).

4E‐BPs (4E‐BP1, 4E‐BP2, and 4E‐BP3) inhibit translation initiation by binding eIF4E and preventing eIF4F formation (Gingras et al. 1999; Lin et al. 1994; Mader et al. 1995; Marcotrigiano et al. 1999; Pause et al. 1994; Poulin et al. 1998; Sonenberg and Dever 2003). mTORC1 phosphorylates 4E‐BPs at Thr37, Thr46, Ser65, and Thr70, inducing their release from eIF4E and enhancing eIF4F complex formation (Bah et al. 2015; Gingras et al. 2001; Lin et al. 1994; Matsuo et al. 1997; Pause et al. 1994; Sonenberg and Gingras 1998). 4E‐BP1 has also been reported to be phosphorylated in vitro by kinases such as cyclin‐dependent kinase 1 (CDK1/CDC2), Protein kinase A (PKA), and MAPK (Fadden et al. 1997; Heesom et al. 2001; Lawrence Jr. et al. 1997; Ramírez‐Valle et al. 2010). However, the relevance of 4E‐BP1/2 phosphorylation by these kinases in the nervous system remains unexplored. 4E‐BPs do not broadly impact general protein synthesis (Colina et al. 2008; Gkogkas et al. 2013; Hooshmandi et al. 2021; Puighermanal et al. 2017; Ran et al. 2013), but rather selectively modulate the translation of specific mRNAs (Choo et al. 2008; Grolleau et al. 2002). In the context of synaptic plasticity and memory, this regulation predominantly operates via the mTORC1–4E‐BP2 axis and eIF4E‐dependent mechanisms (Alasad et al. 2020; Banko et al. 2005; Dowling et al. 2010; Hooshmandi et al. 2021). Target ‘eIF4E‐sensitive’ mRNAs typically possess structured 5′ UTRs or specialized motifs, such as 5′ terminal oligopyrimidine (TOP) tracts (Gkogkas et al. 2013; Hochstoeger and Chao 2024; Hochstoeger et al. 2024; Koromilas et al. 1992; Ran et al. 2013; Thoreen et al. 2012; Truitt et al. 2015).

S6K1/2 are downstream effectors of mTORC1 that promote both translation initiation and elongation. S6K1/2 phosphorylates eIF4B to enhance eIF4A helicase activity, facilitating translation of mRNAs with highly structured 5′ untranslated regions (UTRs) (Dennis et al. 2012; Raught et al. 2004; Rogers Jr. et al. 2001; Shahbazian et al. 2006). mTORC1‐activated S6K1 also phosphorylates and inhibits eukaryotic elongation factor 2 kinase (eEF2K), thereby increasing eEF2 activity and promoting mRNA translation elongation (Wang et al. 2001). Both kinases, particularly S6K2, phosphorylate ribosomal protein S6 at multiple sites (Ser235/236, Ser240/244, and Ser247; Ferrari et al. 1991; Holz and Blenis 2005; Nygård and Nilsson 1990; Pende et al. 2004; Roux et al. 2007). However, genetic studies indicate that these phosphorylation events have minimal impact on the rate of translation, suggesting that S6 phosphorylation may not directly regulate translation (Meyuhas 2015).

The translation of 5′TOP mRNAs is tightly controlled by mTORC1 signaling, directly through the mTORC1 downstream regulator La‐related protein 1 (LARP1) (Fonseca et al. 2015). LARP1 binds the 5′TOP motif to suppress translation, a process modulated by its interaction with RPTOR (Fonseca et al. 2015). Notably, 4E‐BPs exert an indirect yet predominant role in repressing 5′TOP translation (Hochstoeger and Chao 2024; Hochstoeger et al. 2024). Notably, this mode of translation regulation occurs independently of S6K1 activity and S6 phosphorylation (Chauvin et al. 2013; Nygård and Nilsson 1990; Pende et al. 2004; Stolovich et al. 2002; Tang et al. 2001; Thomas 1982). S6K1 and S6 may influence protein synthesis via alternative processes, such as ribosome biogenesis (Dufner and Thomas 1999; Slomnicki et al. 2016).

### 3.2 Translational Regulation of Synaptic Plasticity and Memory by mTORC1

#### 3.2.1 mTORC1 in Healthy Brain Development and Function

mTORC1 signaling in the central nervous system (CNS) exhibits both conserved and unique regulatory dynamics. mTORC1 activity is modulated by metabotropic glutamate receptors (mGluRs), α‐amino‐3‐hydroxy‐5‐methyl‐4‐isoxazolepropionic acid receptors (AMPARs), N‐methyl‐D‐aspartate receptors (NMDARs), insulin receptors, and potassium channels (Gong et al. 2006; Hou and Klann 2004; Raab‐Graham et al. 2006; Wang et al. 2006). PI3K and ERK signaling pathways are major upstream regulators of mTORC1 in the brain (Banko et al. 2005; Briz and Baudry 2014; Kelleher 3rd et al. 2004; Tsokas et al. 2005, 2007). By integrating their signals, mTORC1 supports early brain development by influencing neuronal proliferation, differentiation, migration, polarity, and dendritic architecture (Brown et al. 2012; Paliouras et al. 2012; Shahani et al. 2017; Takei and Nawa 2014; Zou et al. 2011).

Translational control exerted by mTORC1 in the CNS relies predominantly on 4E‐BP1/2. 4E‐BP2 is the principal and most extensively studied paralog in neurons, whereas 4E‐BP1 is largely expressed in glia, and 4E‐BP3 is absent (Aguilar‐Valles et al. 2021; Banko et al. 2005). Phosphorylation of 4E‐BP2 by mTORC1 in the brain relieves its repression of cap‐dependent translation, thereby fine‐tuning synaptic plasticity. However, mTORC1 activity and 4E‐BP2 phosphorylation in the rodent forebrain decline significantly after birth, correlating with a marked reduction in neurogenesis beginning around postnatal day 10–21 (Bidinosti, Martineau, et al. 2010; Bidinosti, Ran, et al. 2010; Encinas et al. 2011; Huang et al. 2024; Kouloulia et al. 2019; Park et al. 2008). Strikingly, deamidation—rather than phosphorylation—is the prominent post‐translational modification of 4E‐BP2 in the brain, in sharp contrast to other organs (Bidinosti, Ran, et al. 2010; Kouloulia et al. 2019). Deamidation at N99/N102 enhances 4E‐BP2's stability by promoting its interaction with the RPTOR–cullin 4B ubiquitin ligase complex while weakening its binding to eIF4E (Bah et al. 2015; Bidinosti, Martineau, et al. 2010; Bidinosti, Ran, et al. 2010; Kouloulia et al. 2019). This modification not only selectively reshapes the neuronal translatome, particularly impacting mRNAs involved in mitochondrial function and nuclear factor κB signaling, but also alters synaptic transmission kinetics (Bidinosti, Ran, et al. 2010; Kouloulia et al. 2019). However, questions remain about the physiological consequences of 4E‐BP2 deamidation across neuronal populations.

In addition to 4E‐BP, mTORC1 also bolsters learning and memory via phosphorylation of S6 through S6K (Antion, Merhav, et al. 2008; Antion, Hou, et al. 2008; Huynh et al. 2014; Pende et al. 2004). S6K promotes mRNA translation in the brain to support stem cell proliferation, growth, and neuronal plasticity processes such as LTP, and mGluR‐LTD (Antion, Merhav, et al. 2008; Antion, Hou, et al. 2008; Kelleher 3rd et al. 2004; Puighermanal et al. 2017; Tsokas et al. 2007). S6K targets eIF4B and eEF2K, promoting mRNA translation elongation in the brain (Eom et al. 2014; Gildish et al. 2012; Im et al. 2009; Narayanan et al. 2008; Shahbazian et al. 2006). Pharmacological activation of mTORC1 by agents like ketamine increases S6 phosphorylation, reversing age‐related impairments in neural stem cell proliferation, thereby enhancing hippocampal neurogenesis in mice (Romine et al. 2015).

Together, these regulators of mTORC1 engage precise and context‐specific mechanisms to orchestrate stimulus‐dependent protein synthesis, which is critical for neuronal health and plasticity throughout life (Costa‐Mattioli et al. 2009; Graber et al. 2013; Hoeffer and Klann 2010; Santini et al. 2014, 2013). However, mTORC1 activation alone is insufficient to induce long‐term synaptic plasticity, suggesting that other protein synthesis‐related mechanisms, such as synaptic tagging, may act in coordination with mTORC1 to support sustained plasticity (Frey and Frey 2008). Nevertheless, a major question remains unresolved: which specific upstream metabolic cues and discrete mRNA subsets are engaged under temporally and spatially regulated conditions within distinct neuronal circuits? Addressing this gap will be vital, given emerging evidence that dysregulation of these finely tuned mechanisms contributes to cognitive deficits and neurological dysfunction (Huang, Mahmood, Psycharis, et al. 2025; Huang, Mahmood, Lacaille, et al. 2025; Huang et al. 2024; Tudor et al. 2016; Zhu et al. 2018).

#### 3.2.2 Pharmacological Modulation of mTORC1 in the Brain

The canonical inhibitor rapamycin has been extensively used to investigate mTORC1's role in neural plasticity. Early work in invertebrate models such as *Aplysia* and *Drosophila* demonstrated that rapamycin blocks long‐lasting synaptic changes (Beaumont et al. 2001; Casadio et al. 1999; Khan et al. 2001; Yanow et al. 1998). Subsequent rodent studies have extended these findings to both hippocampal LTP and LTD (Deli et al. 2012; Fu et al. 2017; Hou and Klann 2004; Stoica et al. 2011; Tang et al. 2002). Specifically, rapamycin disrupts NMDAR‐dependent late‐phase LTP (L‐LTP) in the CA1 region of the hippocampus (Panja et al. 2009; Tang et al. 2002). Rapamycin also blocks dihydroxyphenyl glycine‐induced mTORC1 activation and mGluR‐LTD induction (Hou and Klann 2004; Huber et al. 2000, 2001). Beyond synaptic plasticity, rapamycin disrupts fear memory consolidation and reconsolidation across paradigms and species (Bekinschtein et al. 2007; Blundell et al. 2008; Deli et al. 2012; Gafford et al. 2011; Glover et al. 2010; Jobim et al. 2012; Mac Callum et al. 2014; Parsons et al. 2006; Tischmeyer et al. 2003). However, not all mTORC1 substrates are equally sensitive to rapamycin, as it differentially modulates S6Ks and 4E‐BPs (Choo et al. 2008). Inhibition of 4E‐BP phosphorylation requires more complete inhibition of mTOR kinase activity, which can be achieved with ATP‐competitive inhibitors (e.g., Torin1, PP242) that effectively block phosphorylation at critical sites (at Thr37/46 and Ser65) necessary for eIF4E dissociation (Feldman et al. 2009; Hsieh et al. 2010, 2012; Thoreen et al. 2012, 2009). Among these, Torin1 suppresses TOP mRNA translation and general cap‐dependent initiation in vitro (Thoreen et al. 2012), while PP242 prevents eIF4E hyperactivation and translation‐dependent chemically induced late LTP through 4E‐BP2 (Hsieh et al. 2010; Ran et al. 2013).

Other pharmacological tools, such as 4EGI‐1, which disrupts the eIF4E–eIF4G interaction (Figure 1), revealed that blocking translation initiation impairs fear memory consolidation but spares reconsolidation (Hoeffer et al. 2011). However, co‐administering 4EGI‐1 with the S6K1 inhibitor PF‐4708671 also impairs reconsolidation, underscoring the cooperative role of the axes downstream of mTORC1 in the persistence of associative memory (Huynh et al. 2014).

Overall, pharmacological studies show that mTORC1 selectively controls the translation of mRNAs involved in plasticity and that modulating this pathway may be a promising treatment strategy for neurodevelopmental disorders (NDDs). However, several critical knowledge gaps remain. The unique functions of mTORC1 within specific neuronal populations cannot be fully elucidated using traditional pharmacological approaches. Moreover, investigations into the circuit‐specific contributions of mTORC1 have largely been restricted to the forebrain and basal ganglia regions. Many other brain regions remain understudied, as do the divergent roles of mTORC1 signaling across different developmental stages. Future studies employing temporally controlled pharmacogenetic and optogenetic tools will be critical to addressing these gaps and uncovering the therapeutic potential of mTORC1 modulation in memory disorders and NDDs.

#### 3.2.3 Evidence From Genetic Models

Genetic models targeting mTORC1 signaling components have illuminated its vital role in synaptic function and memory (Table 1). Global KO of its core components (e.g., mTOR, RPTOR, MLST8) is embryonic lethal, necessitating the use of heterozygous (h)KO or cKO models (Cloëtta et al. 2013; Guertin et al. 2006). RPTOR hKO, which inhibits mTORC1, impairs brain development, leading to smaller cell bodies, fewer dendrites, and microcephaly (Zhang et al. 2017); Loss of either upstream inhibitors (e.g., TSC1/2) or downstream translation suppressors (e.g., 4E‐BP2) mimics mTORC1 hyperactivation, causing abnormal dendritic spine morphology, increased spine density, and impaired synaptic formation (Bateup et al. 2011; Cox et al. 2018; Karalis et al. 2022; Niu et al. 2024; Ran et al. 2013; Tavazoie et al. 2005). Interestingly, while both TSC1/2 and 4E‐BP2 KO models show an increased excitatory‐to‐inhibitory synaptic (E/I ratio) input balance, as reflected in elevated miniature excitatory/inhibitory postsynaptic current (mEPSCs/mIPSCs) ratios, the specific synaptic alterations differ between the two models (Bateup et al. 2011; Ran et al. 2013). TSC1/2 loss reduces inhibitory transmission, as evidenced by a reduction in mIPSCs without any change in mEPSCs amplitude. In contrast, 4E‐BP2 deletion increases both excitation and inhibition, as indicated by increased mIPSCs amplitudes and mEPSCs amplitudes and frequencies (Bateup et al. 2011; Benthall et al. 2021; Gkogkas et al. 2010, 2013; Ran et al. 2013; Weston et al. 2014). These findings reveal that complex effects within the mTORC1 pathway differentially regulate synaptic balance and postsynaptic receptor trafficking.

**TABLE 1:** Summary of mTORC1 pathway‐related synaptic plasticity and memory phenotypes in conditional genetic mouse models across diverse neuronal and glial populations.

Protein name	Cell type	mTORC1 activity	Mouse model	Recognition memory			Spatial memory		Associated memory		Working memory	Social memory	Motor learning	Electrophysiology		Publication
				NOL	OPL	NOR	MWM	BM	CFC	Cued FC	T/Y‐maze	SN (SPSN)	Rotarod	LTD	LTP	
TSC	EXs	↑	*NA*													
	INs	↑	*Tsc1* ^*flx+flx*^ *:Dlx5/6‐Cre‐EGFP*													
	INs‐PV	↑	*Tsc1* ^*flx/flx*^ *: Pvalb‐Cre*									↓				Amegandjin et al. (2021)
	INs‐SST	↑	*Tsc1* ^*flx/+*^ *:Sst‐Cre:R26‐EYFP*					Learning: ↑ Memory: ↑	24 h: ↑							Artinian et al. (2019)
	INs‐PCs	↑	*Tsc1* ^*flx/flx*^ *:L7‐Cre*				Reversal learning: ↓ (Water T‐maze)					↓	↓			Tsai et al. (2012)
RPTOR	Full‐body	↓	*Rptor* ^*−/+*^										↓			Zhang et al. (2017)
	EXs	↓	*Rptor* ^*flx/flx*^ *: Camk2a‐Cre*	↔	↔	↔					↓	↔		↔		Wiebe et al. (2023); Zhu et al. (2018); De Gregorio et al. (2021)
	INs	↓	*Rptor* ^*flx/flx*^ *:Gad2‐Cre*	↓	↓	↓					↓	↔				Huang et al. (2024); Wiebe et al. (2023); De Gregorio et al. (2021)
	INs (CPu)	↑	*CAG‐mTOR* ^*SL1+IT/+*^ *:Dlx1‐CreER* ^*T2/+*^								↔		↓			Chen et al. (2024)
	INs‐SST	↓	*Rptor* ^*flx/flx*^ *: Sst‐Cre*					Learning: ↔ Memory: ↓	1 h: ↔ 24 h: ↓	1 h: ↔ 24 h: ↔						Artinian et al. (2019)
4E‐BP2	Full‐body	↑	*Eif4ebp2* ^*−/−*^				↓		1 h: ↔ 24 h: ↓	2 h: ↔ 24 h: ↔	↓				Lowering thresholds for L‐LTP	Banko et al. (2005); Banko et al. (2007); Ran et al. (2013)
	EXs	↑	*Eif4ebp2* ^*flx/flx*^ *:Camk2a‐Cre*	↔	↔	↔			↔^a^			↔				Huang et al. (2024); Wiebe et al. (2019); Huang, Mahmood, Psycharis, et al. (2025); Huang, Mahmood, Lacaille, et al. (2025)
	EXs (LA)	↓	*Opto4E‐BP*; *CamK2α‐Cre*							2 h: ↔ 24 h: ↓						Alapin et al. (2023)
	INs	↑	*Eif4ebp2* ^*flx/flx*^ *:Gad2‐Cre*	↓	↓	↓	Learning: ↔ Memory: ↓^a^		1 h: ↓ 24 h: ↓^a^			↔				Huang et al. (2024); Wiebe et al. (2019); Huang, Mahmood, Psycharis, et al. (2025); Huang, Mahmood, Lacaille, et al. (2025)
	INs‐PV	↑	*Eif4ebp2* ^*flx/flx*^ *:Pvalb‐Cre*	↔		↔			↔							Huang et al. (2024)
	INs‐SST	↑	*Eif4ebp2* ^*flx/flx*^ *: Sst‐Cre*	↔		↔										Huang et al. (2024)
	INs‐VIP	↑	*Eif4ebp2* ^*flx/flx*^ *: Vip‐Cre*	↔		↔										Huang et al. (2024)
	PCs	↑	*Eif4ebp2* ^*flx/flx*^ *:L7‐Cre*				Learning: ↓ Memory: ↓		24 h: ↔			↔	↔	↔		Hooshmandi et al. (2021)
	Glia‐ACs	↑	*Eif4ebp2* ^*flx/flx*^ *: Gfap‐Cre*									↔				Wiebe et al. (2019)
S6K	Full‐body		S6k1^−/−^				Learning: ↔ Memory: 3rd day crossing ↓; 7th day crossing ↔		1 h: ↓ 24 h: ↓ 3 days: ↓	2 h: ↔ 24 h: ↔				↔	E‐LTP: ↓ L‐LTP: ↔	Antion, Merhav, et al. (2008); Antion, Hou, et al. (2008); Koehl et al. (2021)
	Full‐body		S6k2^−/−^				Learning: ↔ Memory: ↔		1 h: ↔ 24 h: ↔ 7 days: ↓	2 h: ↔ 24 h: ↔ 7 days: ↔				↑	E‐LTP: ↔ L‐LTP: ↔	Antion, Merhav, et al. (2008); Antion, Hou, et al. (2008)

*Note:* Effects of both mTORC1 inhibition (e.g., via RPTOR deletion) and hyperactivation (e.g., via TSC1/2 or 4E‐BP2 deletion) are included. Synaptic plasticity outcomes (e.g., E‐LTP, L‐LTP LTD) and behavioral phenotypes are annotated based on cell type specificity. ↑, upregulated/enhanced; ↓, downregulated/impaired; ↔, no significant change.

Abbreviations: 4E‐BP2, eIF4E binding protein 2; ACs, astrocytes; CPu, caudate‐putamen; E‐LTP, early‐phase LTP; EXs, excitatory neurons; INs, inhibitory neurons; LA, lateral amygdala; L‐LTP, late‐phase LTP; LTD, long‐term depression; LTP, long‐term potentiation; PCs, Purkinje cells; PV, parvalbumin‐expressing interneurons; RPTOR, regulatory associated protein of mTOR; S6K, ribosomal protein S6 kinase; SST, somatostatin‐expressing interneurons; TBS_opto_, optogenetic theta burst stimulation; TSC1/2, tuberous sclerosis complex 1 and 2; VIP, vasoactive intestinal peptide‐expressing interneurons.

^a^ Unpublished data.

At the plasticity level, TSC2 hKO and 4E‐BP2 KO mouse models both display enhanced conversion of early LTP (E‐LTP) to L‐LTP, suggesting that removing the translational brakes lowers the threshold for long‐term plasticity changes (Banko et al. 2006, 2005; Ehninger et al. 2008; Ran et al. 2009; Stoica et al. 2011). However, mGluR‐LTD is abolished in TSC1 KO mice but enhanced in S6K1/2 double KO and 4E‐BP2 KO mice, possibly due to feedback relief or selective mRNA translation (Antion, Merhav, et al. 2008; Antion, Hou, et al. 2008; Banko et al. 2006; Bateup et al. 2011; Zhu et al. 2018). Meanwhile, short‐term synaptic plasticity that does not depend on protein synthesis, such as paired‐pulse facilitation, remains unaffected in mice lacking FK506‐binding protein 12 (FKBP12, which forms the rapamycin–FKBP12 inhibitory complex with mTOR), 4E‐BP2, or TSC1/2 in the brain, as expected (Benthall et al. 2021; Ehninger et al. 2008; Hoeffer et al. 2008; Ran et al. 2013; Stoica et al. 2011). These findings demonstrate the selective engagement of mTORC1‐mediated regulation of translation in long‐lasting but not transient forms of synaptic plasticity.

Behaviorally, 4E‐BP2 KO and TSC2 hKO mice display deficits in long‐term spatial and context‐associated fear memory, whereas S6K1 or S6K2 KO do not show spatial learning impairments (Antion, Merhav, et al. 2008; Antion, Hou, et al. 2008; Banko et al. 2005; Ehninger et al. 2008; Koehl et al. 2021). Recent optogenetic studies have linked amygdala‐specific 4E‐BP2 translation to cue‐associated fear memory, reinforcing its role in associative learning (Alapin et al. 2023; Oliveira et al. 2025). Notably, although short‐term memory (STM) is thought to be translation‐independent, deficits in working memory have been observed in models with RPTOR cKO in either excitatory or inhibitory neurons or 4E‐BP2 KO (Banko et al. 2007; Wiebe et al. 2023). These impairments may stem from developmental abnormalities caused by early loss of mTORC1. Finally, the role of 4E‐BP1 in memory remains underexplored. Future studies using temporally precise tools, such as Opto4EBP, may help distinguish between the developmental and acute roles of translation in STM (Alapin et al. 2023; Oliveira et al. 2025).

Taken together, the genetic models reveal how mTORC1 signaling influences neuronal connectivity, synaptic plasticity, and memory. They highlight the distinct contributions of individual mTORC1 regulators to specific types of memory formation and maintenance, reflecting the complex function of mTORC1‐dependent translation regulation in the brain. In the next section, we will discuss the cell type‐specific roles of mTORC1‐mediated translational control in synaptic plasticity and memory.

### 3.3 Cell Type‐Specific Translational Control via mTORC1 and 4E‐BPs in Memory

#### 3.3.1 Studies of Excitatory Neurons Using cKO Models

In excitatory neurons, mTORC1 activity undergoes a significant decline during development, as shown by age‐dependent reductions in S6 and 4E‐BP1/2 phosphorylation (Bidinosti, Ran, et al. 2010; Huang et al. 2024; Kouloulia et al. 2019). Failure to appropriately reduce mTORC1 activity during critical periods can result in profound structural abnormalities: TSC1/2 deletion or mTORC1 hyperactive mutants cause impaired cell migration and enlarged brain size, which are partially reversed by rapamycin treatment (Carson et al. 2012; Ehninger et al. 2008; Kassai et al. 2014; Rozas et al. 2015).

**FIGURE 2:**
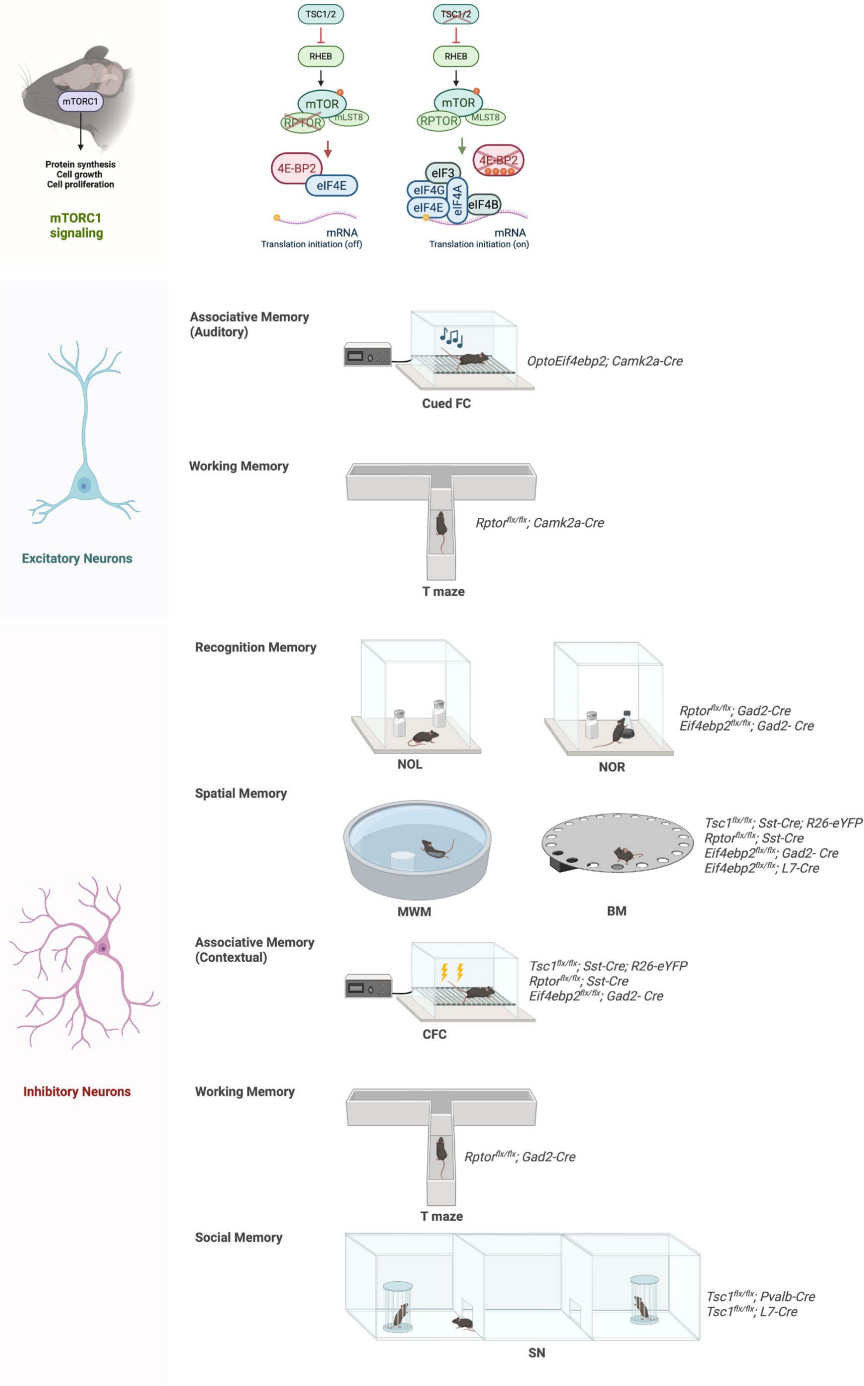
Behavioral changes in memory paradigms following full‐body or cell type‐specific mTORC1 manipulation. Effects of cell type‐specific mTORC1 modulation on the performance of various behavioral tasks commonly used to assess associative, spatial, working, recognition, and social memory in mice. Loss of mTORC1 signaling is modeled by RPTOR cKO, while pathway hyperactivation is induced by deletion of TSC1/2 or 4E‐BP2 or by optogenetic inhibition of 4E‐BP2. Each behavioral outcome is mapped to the targeted cell population using Cre‐driver lines. 4E‐BP2, eIF4E binding protein 2; BM, Barnes maze; CFC, contextual fear conditioning; cued fear conditioning; eIF4F, eukaryotic initiation factor 4F; MLST8, mammalian lethal with SEC13 protein 8; mTOR, mechanistic target of rapamycin; MWM, Morris water maze; NOL, novel object location; NOR, novel object recognition; PCs, Purkinje cells; PV, parvalbumin‐expressing interneuron; RHEB, RAS homolog enriched in brain; RPTOR, regulatory associated protein of mTOR; SN, social novelty; SST, somatostatin‐expressing interneuron; TSC1/2, tuberous sclerosis complex 1/2; VIP, vasoactive intestinal peptide‐expressing interneuron.

In mature excitatory neurons, loss of 4E‐BP2 leads to altered dendritic spine morphology and impaired L‐LTP under physiological stimulation, underscoring the importance of finely tuned mTORC1 signaling for long‐term synaptic plasticity (Ran et al. 2013). However, these mechanisms appear dispensable for mGluR‐LTD and recognition memory, including object‐ and location‐based tasks (e.g., novel object location, object place learning, novel object recognition), which remain intact following deletion of either RPTOR or 4E‐BP2, or following designer receptor exclusively activated by designer drug (DREADD)‐mediated mTORC1 disruption within excitatory neurons (Table 1, Figure 2; Huang, Mahmood, Psycharis, et al. 2025; Huang, Mahmood, Lacaille, et al. 2025; Huang et al. 2024; Tudor et al. 2016; Zhu et al. 2018). These findings suggest that low basal mTORC1 activity in excitatory neurons may not be required for memory under low cognitive loads or weak stimulation protocols. Alternatively, parallel signaling pathways in excitatory neurons may support LTD and LTD‐dependent learning and memory, such as mTORC2, MAPK/ERK, and calcium/calmodulin‐dependent protein kinase II (CaMKII) (Zhu et al. 2018).

Under more demanding paradigms, such as fear conditioning and the Morris water maze, eIF4E‐dependent translational control appears to be more prominently engaged in excitatory neurons (Table 1, Figure 2). For example, contextual fear conditioning induces extensive proteome remodeling in hippocampal excitatory and somatostatin‐positive inhibitory neurons, whereas other interneuron populations appear to fine‐tune synaptic transmission instead (Oliveira et al. 2025). However, our unpublished findings show that deletion of 4E‐BP2 in inhibitory neurons, but not excitatory neurons, impairs contextual fear memory (Huang, Mahmood, Psycharis, et al. 2025; Huang, Mahmood, Lacaille, et al. 2025). This suggests differential proteostatic mechanisms in hippocampal inhibitory versus excitatory neurons. In excitatory neurons, alternative mechanisms of translation initiation and elongation may support synaptic plasticity and memory formation when mTORC is impaired. For example, the excitatory eIF2α and ERK pathways have been implicated in contextual fear memory (Ho‐Tieng et al. 2025; Mahmood et al. 2024; Ryu et al. 2019; Sharma et al. 2020; Simbriger et al. 2021). The eIF2α regulatory pathway, particularly through its kinase PERK, modulates translation and regulates synaptic plasticity (Costa‐Mattioli et al. 2009; Trinh and Klann 2013; Zimmermann et al. 2018). Inhibiting PERK can improve memory performance (Sharma et al. 2018). Moreover, Zimmermann et al. (2018) that PERK deletion reduces eEF2 phosphorylation, thereby enhancing translation elongation and L‐LTP under mTORC1 inhibition (Zimmermann et al. 2018). Furthermore, optogenetic suppression of 4E‐BP2‐dependent translation via Opto4EBP disrupts long‐term cued fear memory, suggesting a temporally and regionally specific requirement for eIF4E‐dependent translation in amygdala excitatory neurons (Alapin et al. 2023). Together, these results highlight a potential division of translational regulation across circuits: mTORC1‐dependent translation may be critical for cued fear learning in amygdala excitatory neurons, whereas in the hippocampus, 4E‐BP2‐mediated translational control in inhibitory gamma‐aminobutyric acid (GABA)ergic neurons and translation regulation via other pathways may be more relevant for contextual fear learning (Table 1, Figure 2). Future studies using cell type‐ and region‐specific tools, such as Opto4EBP and cKOs of TSC1/2, RPTOR, or S6Ks, will be essential to further delineate these circuit‐specific mechanisms of translational control (Alapin et al. 2023; De Gregorio et al. 2021; Wiebe et al. 2019).

#### 3.3.2 Studies of Inhibitory Neurons Using cKO Models

In contrast to excitatory neurons, mTORC1 remains highly active in GABAergic interneurons during early postnatal development (Amegandjin et al. 2021; Huang et al. 2024). Overactivation of mTORC1 via TSC1 cKO in either parvalbumin (PV)^+^ or somatostatin (SST)^+^ interneurons alters their intrinsic firing properties and disrupts inhibitory maturation, underscoring the importance of mTORC1 in interneuron development and circuit formation (Amegandjin et al. 2021; Benthall et al. 2021; Deng et al. 2021; Fu et al. 2012; Malik et al. 2019).

In interneurons, the mTORC1–4E‐BP2 axis appears to be essential for multiple forms of memory. Disruption of this axis in all GABAergic inhibitory neurons, through deletion of either RPTOR or 4E‐BP2, or post‐learning DREADD‐mediated mTORC1 disruption impairs forebrain‐dependent object location and recognition memory (Table 1, Figure 2; Huang, Mahmood, Psycharis, et al. 2025; Huang, Mahmood, Lacaille, et al. 2025; Huang et al. 2024). Our unpublished data indicate that interneuron 4E‐BP2 also plays a crucial role in context‐associated fear memory and spatial memory (Huang, Mahmood, Psycharis, et al. 2025; Huang, Mahmood, Lacaille, et al. 2025). Interestingly, recent studies highlighted the critical role of mTORC1‐mediated plasticity in SST^+^ interneurons in various forms of learning and memory, including recognition memory in object location, goal‐directed spatial learning, and contextual fear‐associated memory (Table 1, Figure 2; Alapin et al. 2023; Artinian et al. 2019; Honoré and Lacaille 2022; Michon et al. 2023). Specifically, SST^+^ interneurons' activity is necessary for object location learning; mTORC1‐mediated LTP at pyramidal‐SST synapses is sufficient to facilitate memory consolidation (Honoré and Lacaille 2022). Goal‐directed spatial learning recruits mTORC1‐dependent reward‐related activity in SST^+^ interneurons of the striatum, suggesting their involvement in encoding reward‐associated locations (Michon et al. 2023). Similarly, fear learning activates mTORC1 in SST^+^ cells, inducing persistent LTP and affecting Schaffer‐collateral plasticity in CA1 pyramidal neurons (Artinian et al. 2019; Oliveira et al. 2025). In addition, loss of either 4E‐BP2 or TSC1 in Purkinje cells (PCs), the principal inhibitory neurons of the cerebellar cortex, causes alterations in intrinsic excitability and spatial memory formation (Table 1, Figure 2; Hooshmandi et al. 2021; Tsai et al. 2012).

Besides these paradigms, motor learning and memory require mTORC1 in the hippocampus, striatum, and cerebellum (De Leonibus et al. 2003; Gerfen and Surmeier 2011; Ghiglieri et al. 2011; Wilhelm et al. 2023). Overexpression of mTOR (via CAG‐mTOR) impairs motor learning, whereas TSC1 deletion in direct but not indirect striatal projection neurons enhances habit formation (Benthall et al. 2021; Chen et al. 2024). Additionally, 4E‐BP2 or TSC1 deletion in PCs impairs performance in motor learning tasks (Hooshmandi et al. 2021; Tsai et al. 2012). These findings suggest that mTORC1 exerts circuit‐specific effects, with hyperactivation promoting or hindering performance depending on the task structure and neuronal subtype.

Despite their lower abundance, inhibitory neurons exert critical control over cognition. Overall, mTORC1‐dependent translation regulation in inhibitory neurons, especially through 4E‐BP2, is essential for long‐term recognition memory, spatial learning, and associated memory, and its dysfunction leads to profound cognitive impairments. However, the precise mechanisms by which mTORC1 signaling in inhibitory neurons shapes memory processes, likely by modulating inhibitory synaptic plasticity and the excitation–inhibition balance, remain poorly defined. Furthermore, the developmental vs adult‐specific functions of mTORC1 signaling in inhibitory neurons also need further delineation, particularly in the context of NDDs and memory disorders. Future studies employing cell type‐specific translatomic profiling will be crucial to identify downstream translational targets of 4E‐BP2 within interneurons and decipher its role in memory regulation.

#### 3.3.3 Studies of Glial Cells Using cKO Models

Though understudied, glial mTORC1 signaling contributes to structural brain integrity, neuroinflammation, and potentially cognitive function. Deletion of TSC1/2 in astrocytes and radial glia causes hippocampal enlargement and pyramidal cell disorganization (Uhlmann et al. 2002; Way et al. 2009). RPTOR cKO in glia induces astrogliosis, implicating mTORC1 in glial responses to injury and activity (Zhang et al. 2017). Recent data suggest that mTORC1 dysregulation in glia contributes to memory deficits and neurodegenerative pathology, particularly in Alzheimer's disease (AD) (Bermudez et al. 2024; Querfurth and Lee 2021).

This emerging evidence suggests that glial mTORC1 signaling modulates synaptic plasticity indirectly through pathways involved in neuroinflammation, metabolic coupling, and myelination. Despite these insights, the behavioral consequences of glia‐specific mTORC1 dysregulation remain poorly defined in the context of neurodevelopmental and neurodegenerative conditions. Future research should prioritize cell type‐specific manipulations to investigate how mTORC1 signaling in astrocytes, microglia, and oligodendrocytes modulates the neuronal microenvironment and affects cognition in disease models. This knowledge will be pivotal to advancing therapeutic strategies that target glial dysfunction as a modifiable contributor to cognitive decline and neurodegeneration.

## 4 Technological Advances for Cell Type‐Specific Research

In the previous section, we summarized recent discoveries on the cell‐type specific role of mTORC1 and translation in learning and memory. These advances have been made possible by powerful technologies—including genetic models, viral vectors, chemogenetic/optogenetic tools, as well as transcriptomic/translatomic approaches, which allow researchers to study translational control across distinct brain cell populations. In this section, we discuss these methods in detail and highlight how they have enabled dissection of mTORC1‐dependent translation regulation in memory processes.

### 4.1 Genetic Systems

The Cre‐loxP system remains the principal tool for cell type‐specific gene manipulation, enabling the generation of cKO mouse models in which loxP‐flanked genes are selectively deleted in distinct cell populations. Such models have provided crucial insights into the cell type‐specific regulation of mTORC1 in memory (Table 1, Figure 2). Neuron‐specific promoters, such as CaMKII subunit alpha (*Camk2a*) and empty spiracles homeobox 1 (*Emx1*), are commonly used to drive Cre expression in forebrain excitatory neurons, while the glutamic acid decarboxylase 2 (*Gad2*) and vesicular GABA transporter (*Vgat*) promoters are used to target genes in GABAergic neurons (Huber and Ernsberger 2006). Using these, Huang et al. (2024) demonstrated that mTORC1 activity in inhibitory, but not excitatory, neurons is essential for hippocampus‐dependent memory (Huang et al. 2024). Interneurons subtype‐specific manipulation of mTORC1 has been achieved using promoters like *Sst*, *Pv*, *Vip*, and *L7* (Amegandjin et al. 2021; Artinian et al. 2019; Hooshmandi et al. 2021; Huang et al. 2024). Other promoters used include choline acetyltransferase (*Chat*), dopamine receptor D1a (*Drd1a*), and preprotachykinin 1 (*Tac1*)—an extensive promoter database is available from the Allen Brain Atlas (http://connectivity.brain‐map.org/transgenic/). However, many of these promoters initiate expression early in development (e.g., *Emx1*: E9.5; *Gad2*: E17; *Chat*: E11), which may confound studies by affecting neuronal development or causing embryonic lethality (Bayer et al. 1999; Dupuy and Houser 1996; Gulisano et al. 1996; Huber and Ernsberger 2006). To address this, tamoxifen (TAM)‐inducible CreER systems are valuable tools to bypass the developmental effects of gene deletion in animals. Here, Cre recombinase is fused to a modified estrogen receptor (Cre‐ERT2) that is activated by TAM (or its active metabolite 4‐hydroxytamoxifen, 4‐OHT) but not endogenous estrogens (Indra et al. 1999). However, optimal TAM dosing is critical as low doses yield incomplete recombination, while high doses can be toxic (Donocoff et al. 2020; Ilchuk et al. 2022).

Viral vectors have become established tools for genetic manipulation in brain cells. These applications include using designed adeno‐associated viruses (AAVs) since they allow for efficient gene delivery, specific nervous cell tropism, and sustained transgene expression. AAVs are used to introduce site‐specific recombinases to control gene expression. The three most common recombinases used are Cre, Flp, and Dre; these enzymes recognize the DNA target sites loxP, FRT, and Rox respectively (Liu et al. 2020). Placing such recombinase genes under the control of cell type–specific promoters within AAVs allows the selective activation or deletion of a target gene in specific neuronal or glial populations. Intracranial delivery of recombinase‐expressing AAVs at chosen developmental or behavioral stages allows for more precise functional dissection of target genes. It should be noted that the choice of AAV serotypes and dose is an important determinant in the efficiency of transduction. For instance, AAV2 and AAV5 exhibit strong neuronal specificity, whereas AAV8 and AAV9 demonstrate broader tropism, efficiently transducing both neuronal and non‐neuronal cell types, with AAV9 notable for its ability to cross the blood–brain barrier (Burger et al. 2004; O'Carroll et al. 2020). Some serotypes like AAV1, AAV5, and AAV8 seem to be involved in gliotoxicity (Howard et al. 2008; Taymans et al. 2007). Therefore, vector selection and the optimization of dosage must be taken into consideration.

### 4.2 Chemogenetic and Optogenetic Tools

Beyond genetic deletions, chemogenetic and optogenetic tools provide spatial and temporal control of neuronal activity. Among these, Designer Receptors Exclusively Activated by Designer Drugs (DREADDs) can indirectly modulate signaling pathways such as mTORC1 (Huang, Mahmood, Psycharis, et al. 2025; Huang, Mahmood, Lacaille, et al. 2025). However, because DREADDs‐induced activation of engineered G protein‐coupled receptors can engage multiple downstream pathways, it is difficult to isolate the specific contributions of mTORC1 or translation per se. The Klann lab has developed several innovative chemogenetic and optogenetic tools that overcome this limitation, providing direct, cell type‐specific control of protein synthesis that enables researchers to dissect its role in memory formation. First, chemogenetically inducible protein synthesis inhibition uses an engineered, Cre‐dependent, drug‐inducible form of the double‐stranded RNA‐activated protein kinase (iPKR) (Shrestha et al. 2020). Upon administration of its inducer, Asunaprevir, iPKR phosphorylates eIF2α at Ser51, a modification that suppresses translation initiation and is reversible within hours after induction. Second, Opto4EBP enables light‐controlled suppression of cap‐dependent translation by activating a modified 4E‐BP2 (with mutations at the rapamycin‐sensitive late phosphorylation sites Ser65 and Thr70), allowing both spatial and temporal resolution (Alapin et al. 2023; Böhm et al. 2021; Dawson et al. 2020). Other optogenetic tools, such as photoactivatable genetically encoded RNA‐binding proteins, also provide reversible control over translation and are promising approaches for future research of cap‐dependent translation processes (Liu et al. 2022; Weissenboeck et al. 2023). Third, shmiR‐*Eif4e* is a short hairpin RNA targeting *Eif4e* embedded in a miRNA backbone and controlled by tetracycline‐responsive elements that enable chemogenetic eIF4E knockdown (Shrestha et al. 2020).

These tools provide the means for acute, reversible, and cell type‐specific manipulation of translation during learning and memory, making them particularly valuable for probing cause and effect relationships in plasticity. Future work should focus on applying these tools to in vivo experiments to dissect cell type and circuit‐specific translation dynamics during synaptic changes, memory consolidation, and pathological conditions.

### 4.3 Transcriptomic and Translatomic Technologies

Transcriptomics allows detailed cell type‐specific analysis of gene expression. Single‐cell RNA sequencing (scRNA‐Seq) has revealed the cellular heterogeneity of the brain and distinct transcriptional signatures across cell types (Eze et al. 2021; Kilfeather et al. 2024; Zeng et al. 2023). By profiling the expression of mTORC1 components in different conditions or in cells lacking mTORC1 components, scRNA‐Seq can help infer how translation is regulated by mTORC1. In addition to scRNA‐Seq, transcriptomic techniques such as cellular indexing of transcriptomes and epitopes by sequencing, which tags cell‐surface proteins alongside RNA profiling, and Patch‐Seq, which links a neuron's physiological properties to its gene expression pattern, enable detailed mapping of brain cell molecular identities and functions (Kumar et al. 2022; Shao et al. 2023).

However, transcriptomic tools only capture transcript abundance, providing no information on translational output. This gap has been addressed by techniques such as translating ribosome affinity purification (TRAP)‐Seq and RiboTag, which enable the isolation of ribosome‐associated mRNAs from genetically defined cell types (Choe and Cho 2020; Heiman et al. 2008). These techniques use genetically tagged ribosomal protein—either L10a (in TRAP) or Rpl22 (in RiboTag)—in different cell types so that any mRNA bound to the ribosomes can be purified and sequenced, thereby providing insights into transcripts that are actively translated in selective cell types. Studies have examined the impact of 4E‐BP1 or 4E‐BP2 deletion in microglia, nociceptors, and interneurons in the context of pain and memory (Bermudez et al. 2024; Lister et al. 2025, 2024; Simbriger et al. 2021; Wong et al. 2023; Huang, Mahmood, Psycharis, et al. 2025; Huang, Mahmood, Lacaille, et al. 2025). It is noteworthy that methods like TRAP‐Seq have limitations. For example, there is a bias as to which mRNAs are captured due to ribosomal heterogeneity and uneven L10a expression, especially when produced via AAV injection (Imai et al. 2023; Shi et al. 2017). TRAP‐Seq also lacks the capacity to distinguish between actively elongating polysomes and stalled ribosomes, and it does not provide codon‐level resolution of ribosome positioning along mRNA transcripts, which is achieved by ribosome footprinting (Ribo‐Seq). In Ribo‐Seq, only the short fragments of mRNA protected by ribosomes are sequenced, enabling single nucleotide mapping of ribosomal positions along transcripts. Although Ribo‐Seq in its standard form is not cell type‐specific, selectivity can be achieved by combining it with TRAP or RiboTag, enabling codon‐level analysis in genetically defined cell populations (Doroudgar et al. 2019; Ingolia 2016; Juntawong et al. 2014).

Many of the above techniques have been applied across various brain cell types. In glial cell studies, fluorescence‐ and magnetic‐activated cell sorting were combined with gene expression analyses to provide robust enrichment of astrocytes, microglia, and oligodendrocytes (Jeffries et al. 2021; Li et al. 2025). It should be noted that microglia may undergo rapid morphological and proteomic changes in vitro and ex vivo. These alterations are influenced by environmental and experimental factors, including nutrient composition and growth factor supplementation, which impact mTORC1 components (e.g., mTOR) and its upstream and downstream effectors (e.g., RHEB, eIF4E) (Lloyd et al. 2024). Such changes can significantly affect the interpretation of mTORC1‐related signaling pathways and should be accounted for when analyzing studies of glial mTORC1 function.

The ability to control and monitor protein synthesis at the level of specific brain circuits will be crucial to resolving the complexity of translational regulation in memory and identifying precise therapeutic targets in cognitive disorders.

## 5 Dysregulation of Translational Control in Neurological Disorders

### 5.1 Dysregulated mRNA Translation in Neurodevelopmental Disorders

NDDs emerge during development and result in impairments that disrupt daily activities and overall quality of life (Morris‐Rosendahl and Crocq 2020). The Diagnostic and Statistical Manual of Mental Disorders (Fifth Edition) includes a comprehensive list of NDDs, including autism spectrum disorder (ASD), intellectual disability, attention deficit/hyperactivity disorder, and several distinct learning and motor disorders (American Psychiatric Association and American Psychiatric Association 2013). Dysregulated mRNA translation, including alterations in mTORC1–4E‐BP2 activity, has been implicated in ASD‐like behaviors (Hooshmandi et al. 2020). Mutations in the genes encoding various factors that regulate translation (eIF4E, eIF4G1, GRB10‐interacting GYF protein 1 (GIGYF1), GIGYF2, and zinc finger protein 598) were identified in people with autism (Simons Foundation Autism Research Initiative, SFARI database). In addition, mutations in *TSC2* (but not *TSC1*) have a propensity to cause cyst‐like tubers that are associated with ASD (Numis et al. 2011). Moreover, heterozygous deletion of either *Tsc2* alone or both *Tsc1* and *Tsc2* induces ASD‐like phenotypes in mice (Kashii et al. 2023). Notably, homozygous deletion of either *Tsc1* or *Tsc2* is embryonic lethal (Kobayashi et al. 2001; Rennebeck et al. 1998).

Fragile X syndrome (FXS) is the most common monogenic cause of ASD. It results from a deficiency of fragile X messenger ribonucleoprotein (FMRP), an RNA binding translational repressor encoded by *FMR1* (Hagerman et al. 2017; Schaeffer et al. 2003). Loss of FMRP in patients with FXS and *Fmr1* KO mice leads to an increase in general mRNA translation due to hyperactivation of mTORC1 and ERK signaling, resulting in FXS pathology (Darnell et al. 2001; Osterweil et al. 2010; Sharma et al. 2010). As a result, studies have suggested that targeting mTORC1 is a therapeutic strategy for FXS. Genetic reduction of S6K1 reversed exaggerated LTD, abnormal neuronal morphology, and motor and memory deficits in *Fmr1 KO* mice (Bhattacharya et al. 2012). Treatment with the small molecule 4EGI‐1, which inhibits the eIF4E‐eIF4G interaction, improved memory deficiencies and abnormal mGluR‐LTD in *Fmr1* KO mice (Santini et al. 2017). Metformin, a common antidiabetic drug, has been shown to improve core deficits in a FXS mouse model (Choi et al. 2024; Gantois et al. 2017), by restoring proper eIF4E phosphorylation. Based on the success of metformin in correcting core FXS phenotypes in preclinical settings, several clinical trials (NCT03862950, NCT03479476) are either ongoing or recently completed. If successful, this strategy could significantly enhance the quality of life of individuals with FXS. Moreover, parent‐implemented language intervention alone or in combination with lovastatin (which can inhibit mTORC1) significantly improved behavioral outcomes in patients with FXS (Thurman et al. 2020). Nevertheless, these findings indicate the therapeutic promise of normalizing dysregulated translation by modulating mTORC1 in FXS and potentially other ASD‐related disorders.

A recent study demonstrated that mutations in KICSTOR subunit 2 (*KICS2*) affect the KICSTOR (KPTN, ITFG2, C12orf66, and SZT2 regulatory) complex, which negatively regulates mTORC1 signaling, resulting in intellectual disability (Buchert et al. 2025). This disruption activates mTORC1, resulting in impaired ciliary function that is implicated in epilepsy and psychiatric comorbidities (Liu et al. 2021). Indeed, hyperactivation or overexpression of mTOR is a defining molecular feature of a class of neurodevelopmental epilepsies (Crino 2020). These data consolidate mTORC1‐dependent translation as a pivotal pathway in neurodevelopmental pathophysiology and a compelling target for intervention.

### 5.2 Neurodegenerative Diseases

AD, the most common neurodegenerative disorder, is characterized by the accumulation of amyloid β (Aβ) plaques and the formation of neurofibrillary tangles caused by hyperphosphorylated tau (Pradeepkiran et al. 2024). These pathological features contribute to neuronal cell death and subsequent loss of synaptic connections, resulting in cognitive impairment (Ittner and Götz 2011). Post‐mortem analyses revealed mTORC1 hyperactivation in the hippocampus and inferior parietal lobule, evidenced by elevated phosphorylation of mTORC1 components including mTOR, RPTOR, and S6K (Sun et al. 2014; Tramutola et al. 2015). It has been demonstrated that mTORC1 hyperactivation indirectly affects tau phosphorylation in mice, indicating a contribution to neurodegenerative processes (Caccamo et al. 2013). Activation of mTORC1 inhibits autophagy, a critical pathway involved in clearing Aβ and other misfolded proteins (Li et al. 2017). Preclinical studies indicate that treatment with mTOR inhibitors such as rapamycin can improve cognitive impairments associated with AD (Barbour et al. 2024; Ding et al. 2021). Other mTOR inhibitors, such as OSI‐027, AZD2014, and AZD8055, have shown promising effects in reducing tau levels (Silva et al. 2020). However, the role of mTORC1 signaling in AD is complex, with conflicting findings regarding its impact. Some studies report that suppressing mTOR signaling reduces amyloid pathology as mTORC1 activity is often elevated in the AD brain (Caccamo et al. 2015, 2014). Meanwhile, other work suggests that in AD models, inhibiting mTORC1 impairs LTP (Ma et al. 2010). More recently, depletion of translation repressors 4E‐BP1/2 (akin to hyperactivated mTORC1 and 4E‐BP1/2 hyperphosphorylation) in microglial (BV2) cells mitigates Aβ oligomer toxicity, and 4E‐BP2 hKO preserves cognition in AβO‐infused mice (Bermudez et al. 2024; Ribeiro et al. 2024). Future research could prioritize cell type‐specific manipulations to investigate how mTORC1 signaling in glial cells affects cognition in disease models. Particular attention should be given to 4E‐BP1, which is predominantly expressed in glial cells and has been shown to be upregulated in Alzheimer's disease models (Baleriola et al. 2014; Bermudez et al. 2024; Gouveia Roque et al. 2023; Michopoulou et al. 2022). These findings imply a complex role of translation initiation in AD pathophysiology and indicate the need for further investigation into its cell‐type specific functions.

In Parkinson's disease (PD), α‐synuclein accumulation generates Lewy bodies, which disrupt dopaminergic neurons, leading to impaired motor activity (Srinivasan et al. 2021). Lewy bodies engender neurotoxicity by disrupting the TSC1/2 complex, resulting in mTORC1 hyperactivation and aberrant mRNA translation (Khan et al. 2023). Post‐mortem PD brains exhibited reduced TSC1/2 levels, and pharmacological inhibition of mTORC1 promotes the clearance of α‐synuclein (Bové et al. 2011). Mutations in *EIF4G1* were reported in familial PD (Chartier‐Harlin et al. 2011); however, their frequency and clinical relevance remain debatable due to their rarity and limited functional evidence (Blanckenberg et al. 2014; Lesage et al. 2012; Nishioka et al. 2014). 4E‐BP1 overexpression enhances the mitochondrial unfolded protein response and suppresses PD‐linked pathogenic insults (Dastidar et al. 2020). Using chemogenetic tools or genetic mouse models targeting midbrain dopaminergic neurons to modulate 4E‐BP1/2 levels may provide valuable insights into the mechanisms underlying PD (Bäckman et al. 2006; Lin et al. 2012). Dysregulated mTORC1 signaling is also observed in other neurodegenerative diseases: amyotrophic lateral sclerosis, frontotemporal dementia, and Huntington's disease, as described in detail in a recent review (Querfurth and Lee 2021).

### 5.3 Psychiatric Disorders

Major depressive disorder (MDD) is a highly prevalent psychiatric condition primarily characterized by mood disturbances, which may be accompanied by cognitive impairments (Baxter et al. 2014). Various pharmacological treatments are available, including selective norepinephrine or serotonin reuptake inhibitors, and tricyclic antidepressants; however, 30%–40% of those with MDD do not respond to them (Kupfer 2005; Rizvi et al. 2014; Warden et al. 2007). Ketamine, an NMDAR antagonist, produces fast‐acting antidepressant effects and is approved in the United States and Canada for treatment‐resistant MDD (Lewis et al. 2023). A key mechanism underlying ketamine's effects involve activating the mTORC1–4E‐BP signaling pathway (Aguilar‐Valles et al. 2021; Li et al. 2010; Zhou et al. 2014). 4E‐BPs in inhibitory interneurons mediate the ketamine action in mouse models, as their deletion from inhibitory neurons impacts the depression behavioral response to ketamine and affects synaptic plasticity (Aguilar‐Valles et al. 2021). This suggests that mTORC1‐mediated mRNA translational control may be crucial to treating MDD.

## 6 Conclusions and Prospective

The recognition of mTORC1 signaling and eIF4E‐dependent translational control as critical regulators of synaptic plasticity and memory formation has broadened our understanding of biological processes and pathological dysregulation in the brain. With advances in gene editing technologies, it is now possible to correct mutations in key translational regulators at the genomic level. Furthermore, RNA therapeutics, including antisense oligonucleotides and RNA interference, are being developed to target specific mRNA transcripts for degradation or translation repression, offering highly specific treatment strategies for diseases involving dysregulated protein synthesis. Despite these promising advances, several questions remain. Persistent gaps in our understanding include the cell type‐specific regulation of mTORC1 signaling and the selective control of mRNA translation by 4E‐BPs within diverse neuronal and glial populations, particularly in disease. Moreover, the precise temporal windows during which protein synthesis is necessary to support distinct types of memory formation remain poorly understood, limiting our ability to determine the best timepoints for interventions. Addressing these questions will require applying cell type‐resolved transcriptomic and translatomic profiling and temporally and regionally controlled genetic manipulations in various disease‐relevant models. Future research investigating these translational mechanisms could inform the design of novel targeted therapies for various neurodevelopmental and neurodegenerative disorders.

## Author Contributions

**Ziying Huang:** writing – review and editing, writing – original draft, conceptualization, visualization. **Niaz Mahmood:** writing – review and editing, writing – original draft. **Shane Wiebe:** writing – review and editing. **Arkady Khoutorsky:** writing – review and editing. **Jean‐Claude Lacaille:** writing – review and editing. **Nahum Sonenberg:** writing – review and editing, funding acquisition, supervision.

## Consent

The authors have nothing to report.

## Conflicts of Interest

The authors declare no conflicts of interest.

## Data Availability

The authors have nothing to report.
